# Systemic factors for enhancing intersectoral collaboration for the operationalization of One Health: a case study in India

**DOI:** 10.1186/s12961-021-00727-9

**Published:** 2021-05-04

**Authors:** Sandul Yasobant, Walter Bruchhausen, Deepak Saxena, Timo Falkenberg

**Affiliations:** 1 Center for Development Research (ZEF), University of Bonn, Genscherallee 3, 53113 Bonn, Germany; 2 Global Health, Institute for Hygiene and Public Health (IHPH), University Hospital Bonn, 53127 Bonn, Germany; 3 Indian Institute of Public Health Gandhinagar (IIPHG), 382042 Gandhinagar, India; 4 Datta Meghe Institute of Medical Sciences, Jawaharlal Nehru Medical College, 442004 Wardha, India; 5 GeoHealth Centre, Institute for Hygiene and Public Health (IHPH), University Hospital Bonn, 53127 Bonn, Germany

**Keywords:** Intersectoral collaboration, One Health, Operationalization, Health system, India

## Abstract

**Background:**

One Health is a collaborative, multisectoral, and transdisciplinary approach—working at the local, regional, national, and global levels—with the goal of achieving optimal health outcomes recognizing the interconnection between people, animals, plants, and their shared environment. Operationalization of the One Health approach is still unclear for various local health systems with their respective targets. In this scenario, the empirical study of intersectoral collaboration between the human and animal health systems provides an opportunity to investigate the appropriate strategies and their enabling factors at the local health system level. Thus, this study documented and validated the innovative strategy for intersectoral collaboration, focusing on effectual prevention and control of zoonotic diseases with its enabling factors for a city in western India, Ahmedabad.

**Methods:**

This case study was conducted in three phases: phase I (qualitative data collection, i.e., vignette interview), phase II (quantitative data collection through modified policy Delphi), and phase III (participatory workshop). The vignette data were handled for content analysis, and the Delphi data, like other quantitative data, for descriptive statistics. The participatory workshop adapts the computerized Sensitivity Model^®^ developed by Vester to analyse the health system dynamics.

**Result:**

Out of the possible 36 strategies, this study validated the top 15 essential (must-have) and five preferred (should-have) strategies for the study area. For operationalization of the One Health approach, the enabling factors that were identified through the systems approach are *micro-level factors* at the individual level (trust, leadership, motivation, knowledge)*, meso-level factors* at the organizational level (human resource, capacity-building, shared vision, decision-making capacity, laboratory capacity, surveillance)*, macro-level factors* at the system level (coordinated roles, relationships, common platform), and *external factors* outside of the system (guidelines/policies, community participation, a specific budget, political will, smart technology).

**Discussion:**

This study reveals that the micro-level factors at the individual level are potential levers of the health system. More attention to these factors could be beneficial for the operationalization of the One Health approach. This study recommends a systems approach through a bottom-up exploration to understand the local health system and its enabling factors, which should be accounted for in formulating future One Health policies.

**Supplementary Information:**

The online version contains supplementary material available at 10.1186/s12961-021-00727-9.

## Introduction

One Health (OH) is a collaborative, multisectoral, and transdisciplinary approach—working at the local, regional, national, and global levels—with the goal of achieving optimal health outcomes, recognizing the interconnection between people, animals, plants, and their shared environment [1]. The recurrent (re-)emergence of zoonotic diseases underscores the action required on the OH concept [2–4]. OH encourages inter-, multi-, and/or transdisciplinary actions, which require the collaboration among various actors in dealing with disease control or risk mitigation and promoting the health, well-being of humans, animals, and the environment [2–4]. OH is an emerging concept; still, it is an amorphous entity within a state of flux, as the OH and its operationalization are experiencing some bridging factors and are impeded by barrier factors [5, 6]. The operationalization of OH involves multiple challenges, such as a lack of policies/guidelines on information and/or resource sharing, biased funding, and imbalanced participation across different sectors [7–9]. To date, OH implementation is recognized as highly politically driven [5] with a top-down approach [10, 11] and with few community-driven initiatives [12, 13]. This top-down approach has its disadvantages in the policy process, such as effectiveness with respect to acceptability, local adaptation, and dynamics of change [14]. In response to the perceived weakness of the top-down perspective, the bottom-up approach [15] provides a platform to analyse the multitude of actors who interact at the operational (local) level on a particular issue, with specific reference to problem-solving [16–19], which might contribute to the sustainable operationalization of OH.

In the absence of a global criterion, intersectoral collaboration (ISC) is one of the key aims for the operationalization of OH [9, 20–22]. Few ISC strategies have been evinced in African [6, 23], Arctic [24], American [25], Asian [26–28], European [29], and Oceanian [30] countries; however, it has been suggested as a means to develop strategies focused on improving the health system structure and its dynamics. To date, there is no such national One Health policy or guideline established for India; thus, an effort to develop strategies and identify enabling factors for improved operationalization can provide evidence to this end. Considering the complexity of the Indian health system, systems thinking principles, where the system and its respective context are viewed as a complex of interrelated and interdependent parts, provides an opportunity to address the above gap [31, 32]. Systems thinking is also being recommended for health system strengthening by WHO, even without an OH ambition [33], which indicates the need for a systems approach to tackle health challenges, as evinced in the literature [34–36]. Within the health system, systems thinking aids in addressing complex health challenges by facilitating the testing of new ideas in the respective systems [31]. With complex adaptive systems thinking principles, this study does not intend to provide an “easy answer” for an ideal ISC for the OH approach. However, it provides a way to consider and cultivate different possible solutions in a context that avoids the “common unintended mishaps” resulting from enforcing linear “expert solutions” [37]. To address this gap, this study adopts the bottom-up approach with the principles of system thinking. This case study aims (1) to document and validate the innovative strategies for ISC, focusing on OH operationalization in the prevention and control of zoonoses, and (2) to document the enabling factors to boost the ISC between the human and animal health systems through a mixed-method approach.

## Materials and methods

### Study design

This case study was conducted in three phases from July to October 2019. In phase I, qualitative data through vignette interviews were collected, followed by quantitative data collection through a modified policy Delphi method in phase II. Phase III collected information through a participatory workshop. This case study is part of a larger health system study executed in India, i.e., the RICOHA (Research to explore intersectoral collaboration for One Health approach) study. The detailed RICOHA study methodology is described elsewhere [38].

### Study sampling

Mixed sampling was applied in this study. For phase I, purposive sampling was used to select the key actors at the local, state, and national levels. A total of eight actors (experts at the state/national level from both the human and the animal health systems) were interviewed after their consent of participation. Out of eight actors, there were two from the local level (one human health, one animal health), two from the district level (one human health, one animal health), two from the state level (one human health, one animal health), and two from the national level (one human health, one animal health). The sample for phase II was drawn from a larger sample of experts. The experts included researchers, academics, policy-makers, and health managers, irrespective of their professional experience level, working at the local, state, or national level. Initially, a large volume of experts (297) was approached, but only one-third provided consent for participating in the policy Delphi survey (even after two reminders). In the end, 23 experts (nine from the local level and 14 from the state/national level) participated in the survey (10 from animal health and 13 from human health). For Phase III, purposive sampling was adapted through a facilitated consultative process to recruit the stakeholders from the local health system level. Both the government and the private institutions working in the domain of the human and animal health systems were identified. The respective departments nominated the appropriate individuals for the workshop. This process was carried out 2 months before the actual date of the workshop. Among others, the participants were: epidemic officer, medial officer of health, surveillance officer from the human health system, zoo veterinarian, superintendent of cattle nuisance control department, foot and mouth disease laboratory director, animal husbandry department director, lead private practitioner, and environmental specialist.

### Data collection and analysis

In phase I, information (Additional file 1) was collected through vignette interviews. This method has been used in clinical [39] and public health settings [40] to solve complex issues. In simpler terms, the vignette technique is a method that can provoke and synthesize perceptions or opinions from the respondents [41]. A semi-structured vignette interview guide hypothesized innovative convergence strategies among the health system actors, and face-to-face interviews were administered with the sampled stakeholders. Interviews were conducted at the date and time convenient to participants. The interviews were recorded after obtaining the participant's consent, and verbatim notes were also taken during the interview. The vignette responses were handled like other qualitative data. Content analysis (inductive) was used to gather proposed strategies from the transcripts. The findings were reported using the consolidated criteria for reporting qualitative research [42] utilizing the software ATLAS.ti version 8 [43].

All the codes (in the form of strategies) derived from the phase I analysis were clustered into themes and presented in phase II. During this phase, information (Additional file 2) was collected through the policy Delphi technique (developed at the RAND Corporation in the 1950s [44]) with health system experts). Through this process, we identified a wide range of validated options and solutions to the respective strategies [45, 46]. An online platform, i.e., Survey Monkey software [47], was used to develop the survey, and potential health system experts were invited via email for participation. The health system experts were asked to rank the importance of each item on a four-point Likert scale: 1: somewhat preferable; 2: very much preferable; 3: somewhat essential; 4: very much essential. The difference between the essential and preferable criteria was explained to the participants. If the presence of a strategy is a “must” within the system to uphold the system's resilience, then the strategy is considered essential, whereas strategies that make the system better but without which the system could also function, are considered as preferable. There was a high nonresponse rate in the first round (about two-thirds) and the second round (about half). The Likert score was utilized to categorize the strategies into essential (must-have) or preferable (should-have) strategies. The cut-off value was set at a level of 60%; if 60% of actors agreed to a strategy being either essential or preferred, then that strategy was considered under the respective category.

For Phase III (Additional file 3); a computerized Sensitivity Model^®^ developed by Vester was adapted in a one-day participatory workshop. This software has its foundation in cybernetics and dealing with complex systems in an interconnected approach [48]. This model facilitates the consensus-building process, based on fuzzy logic reasoning, among participants for a particular issue [49, 50]. This follows a flexible and iterative process with consensus building at a certain level (with repeated deliberation) and minimizes the participant's personal importance. This stemmed into a comprehensive, deterministic, and aggregated outcome at the end of the participatory workshop. The outcome of this participatory workshop provided a comprehensive description of the interactions of factors with their interlinkages in the health system. The workshop was conducted in a stepwise manner as per the Vester model. First, the health system's boundaries, system factors, and representativeness through system viewpoints were discussed, and the criteria matrix was developed. Then, the system factor interlinkages and their roles in the system were allocated, which resulted in the consensus matrix.

A participatory discussion about the health system issues pertaining to OH (especially for zoonoses prevention and control in the local context) was initiated to engage the participants, which was guided by the facilitation process. The main focus was to summarize the problems and especially to understand the subsystems (such as human and animal health, public and private) within the larger system. Some of the discussions were also about the levels of the health system involved, emphasizing the power relations at the national, state, district, corporation, and operational levels. Lead questions, like What are the factors?; How does the system function with or without these factors?; What could be done?, facilitated the process of engagement. From this iterative discussion process, a set of factors with their characteristics was collected and presented for open discussion.

The criteria matrix was developed by assigning a fully, partly, or not applicable criterion to each factor. Values of 1, 0.5, or 0 were for assigned for each criterion, respectively. All system factors were checked for completeness (assessed by all the 18 criteria) from multiple perspectives. The seven levels of consideration covered the key components of the system with three entities. The relations of four aspects of the dynamics and four types of factors to the system resulted in the 18 criteria to weigh the factors (the details on weighing criteria are shown in Additional file 3). The total score of each factor after weighing was compared with each other, and the distribution was discussed from the system viewpoint.

To develop the consensus effect matrix, two representative groups of participants were formed along with one facilitator for guiding the discussions and amending any methodological error. As the main aim was to understand the strength of the factors’ connection and interaction with all other components of the system, a scale of disproportionally strong (3), medium (2), or weak (1) connection or no connection (0) was used. The focus of this scale was only on the strength of interaction, not the direction. The number entered is the one on which the group agreed after a certain amount of thought and discussion. Then, the results of the two groups were compiled, debated, and discussed, and the final score for each pair of factors was agreed upon, forming the final consensus effect matrix.

The sum of horizontal rows from the matrix was calculated as the active sum for each factor *i* (AS*i*), i.e., how strongly a factor affects the rest of the system. Similarly, the sum of the vertical columns was calculated as the passive sum for each factor *i* (PS*i*), i.e., how susceptible a factor is to changes in the system and how it would react to them. In summary, the total effect of a given factor was expressed by the AS*i*, whereas the PS*i* was expressed as the system's total effect on a given factor. To derive the P-value, the AS*i* and PS*i* were multiplied, and to derive the Q-value, the AS*i* and PS*i* were divided.

Based on the P-value (interconnectedness) and the Q-value (impact strength), all the factors were assigned a role in the system. A factor was called critical when the P-value was high, i.e., the factor could influence others in the system and is highly interconnected. The reverse, low P-value, was called buffering [48]. With the help of the Vester system model, these values were plotted (x-axis: PS and y-axis: AS; P-values from the bottom left to the top right and Q-values from the bottom right to top left) and used for the visualization of each factor. The role of each factor within the system was synthesized based on the location of the factor, i.e., active (top left), reactive (bottom right), critical (top right), and buffering (bottom left).

## Results

### Thematic OH strategies derived from the vignette (Phase I)

The content analysis indicated 36 different strategies categorized into themes such as legal or policies, clinical aspects including disease-specific ones, collaborations at the managerial level, collaborations at the provider level, collaborations at the community level, and the inclusion of private actors.

### ISC strategies for the operationalization of OH (Phase II)

Out of 36 different strategies, the top 15 validated must-have, i.e., essential, strategies and the top five validated should-have, i.e., preferred, strategies based on the outcome of the policy Delphi process are presented in Table 1.

**Table 1:** Top fifteen essential strategies and top five preferred strategies validated through the modified policy Delphi process for the operationalization of One Health in the prevention and control of zoonotic diseases in Ahmedabad, India

Essential One Health strategies	Cross-sectoral information and data sharing is recommended within the human and animal health system with an emphasis on the joint data analysis and an early alert system for zoonoses (1, 2, 3, 4, 6, 7, 10, 11, 13, 14, 18) Public health act or clinical establishment act for all the clinics (human/animal) in the city emphasizing reporting diagnosed conditions to the public health system (4, 6, 7, 9, 10, 14, 18) Strengthening the local capacity of laboratories for screening and diagnosis of zoonotic diseases (6, 9, 15, 16) Developing guidelines for disposal of all dead animals irrespective of disease condition for the city (1, 4, 6, 11, 12, 15) Enhancing and strengthening the prophylactic vaccination of all types of animals, especially for rabies prevention (1, 2, 5, 6, 7, 8, 10, 11, 14, 15, 16, 17, 18) Promoting better hygiene and preventive practices among the community, especially for flu prevention (1, 4, 5, 6, 7, 11, 14, 17, 18) Resource sharing with the human/animal health system for improving service delivery and establishing surveillance (1, 2, 3, 5, 6, 7, 8, 11, 12, 13, 14, 16, 18) The reporting pattern for prioritized zoonotic conditions should be established, and regular monitoring of the same is recommended (4, 5, 6, 9, 10, 11, 14, 16, 17, 18) Sharing of knowledge among the medical and the veterinary professions through a common platform, including the joint training programs (1, 4, 7, 13, 14, 16, 18) A common One Health clinical body that is answerable for every situation related to zoonoses management and its prevention (1, 4, 5, 6, 7, 8, 11, 13, 14, 15, 16, 18) Developing informed education and communication (IEC) materials for zoonoses prevention across the clinical setting of both systems to educate their respective patients (4, 13, 16, 17, 18) Cross-communication among the frontline workers at the grass-roots level and cross-sectoral information sharing with appropriate officials for any abnormal occurrence (4, 5, 16, 17, 18) Sensitization of the community along with knowledge and awareness on prevention and control of zoonoses (4, 5, 6, 11, 14, 16, 17, 18) Formulation of a One Health community cell at the grass-roots level with help of frontline health workers and community members (4, 5, 6, 11, 14, 16, 17, 18) Financial incentive packages for the inclusion of private providers into the public health delivery system and for reporting the symptoms and/or diagnosed zoonotic conditions to the system (4, 6, 8, 9, 10, 11, 14, 16, 18)
Preferred One Health strategies	Urban zoonoses and/or One Health committees, like at the district and state level, should be developed for the city level (1, 2, 3, 4, 5, 6, 7, 8, 11, 12, 13, 14, 15, 16) The city should develop animal treatment centres and hostel facilities where stray animals can be inspected and vaccinated regularly (4, 5, 6, 11, 14, 16) In the clinical and primary healthcare setting, a detailed history taking for a provisional diagnosis of zoonotic conditions should be emphasized (4, 6, 8, 13) Financial incentives to animal handlers to report any disease or any abnormal condition(s) of their animals to the public health system (4, 10, 17) Enhancing collaboration among professional bodies like the Indian Medical Association, Indian Veterinary Association, etc. (1, 6, 7, 13)

Numbers in parentheses indicate the serial numbers of factors (see Table 2) responsible for the respective strategy

### Enabling factors for strengthening ISC and OH operationalization (phase III)

The workshop participants defined 18 factors encompassing micro-level factors (at the individual level), meso-level factors (at the organizational level), macro-level factors (at the system level), and external factors (beyond the boundary of the system) of fulfilling the above-mentioned 15 essential strategies for the case of Ahmedabad, India (Table 2). The boundary refers to the local health system comprising human and animal health as controlled by the municipal governments. The set of factors synthesized during the first step of the workshop offers an accumulated and comprehensive perspective about OH's operationalization with a focus on zoonoses prevention and control. As described in the methods, the system boundaries for the OH approach were defined as per the participating stakeholders. The set of factors from a health system viewpoint was confirmed during the workshop's deliberation and discussion phase and cross-checked during the further steps of the workshop.

**Table 2:** Factors for operationalization of One Health in the prevention and control of zoonotic diseases in Ahmedabad, India, extracted from the system workshop during September 2019

Context	Factors	Description
Micro-level factors (individual level)	Leadership quality (1)	Each individual within their sector should take the lead as per their expertise
	Building trust (2)	Trust among the sectors needs to be facilitated for collaborative work
	Motivation for teamwork (3)	Actors should have motivation towards working as a team
	Adequate knowledge (4)	Adequate knowledge of zoonotic conditions for early detection and experiences
Meso-level factors (organizational level)	Adequate human resources (5)	Multidisciplinary team One Health Cell consisting of a representative from a different sector or dedicated human resource within each department for OH
	Capacity-building (6)	Appropriate interprofessional education needs to be targeted towards medical and veterinary education and other clinical experiences for health workers
	Shared vision and objectives (7)	Departmental visions need to be shared with other sectors to form a comprehensive agenda
	Improving decision-making capacity (8)	Capacity-building to take an appropriate decision during the health emergencies and other relevant conditions
	Improving laboratory capacity (9)	Availability of screening and diagnosing zoonotic conditions
	Strengthening surveillance system (10)	The current surveillance system needs to be strengthened. Individual systems should also effort to capture the symptoms from the animals and do a prediction of disease transmission
Macro-level factors (system level)	Coordinating roles (11)	Specific coordinating responsibilities of actors at a different level
	Relationships among actors (12)	A good relationship among staff members should be there irrespective of hierarchy within the respective department
	Common platform (13)	A common platform is necessary to share the knowledge and experiences and could act as a bridge
External factors (beyond the system boundary level)	Structured guidelines/policy (14)	Guidelines on roles and responsibilities of each actor, including the type of activities
	Political will (15)	Both urban and rural governance systems need to work collaboratively. The political commitments need to be enforced with the current system
	Specific budget head (16)	Budget head for specific One Health activities
	Community participation (17)	Community engagement and participation is essential for promoting disease awareness
	Smart technology (18)	Both systems should be able to use smart technologies to share the data and information at any point of time

The numbers in parentheses indicate the serial number of factors

Table 3 presents the final consensus effect matrix (summary of all the factors with their AS and PS values) after the deliberation of two subgroups. A high AS value, as attributed to adequate knowledge (4), signifies the high influence on the others in the system, whereas a low AS value, e.g., community participation (17), signifies low influence and requires an extensive change to influence the system. Similarly, other factors of the system influence the factors with high PS value, e.g., strengthening surveillance system (10). In contrast, a low PS value, e.g., motivation for teamwork (3), indicates that extreme system changes are necessary to affect the factors.

**Table 3:** Consensus matrix representing the strength of the direct effects among factors extracted from the system workshop for the operationalization of One Health during September 2019

Influenced by↓/to →	(1)	(2)	(3)	(4)	(5)	(6)	(7)	(8)	(9)	(10)	(11)	(12)	(13)	(14)	(15)	(16)	(17)	(18)	AS	P
(1)	X	2	2	2	3	3	2	3	0	1	3	1	2	2	3	0	1	0	30	540
(2)	2	X	2	2	2	2	3	2	2	2	3	2	3	2	1	0	2	0	32	384
(3)	2	1	X	0	2	3	2	2	0	2	3	2	3	1	0	0	2	0	25	375
(4)	1	2	2	X	2	3	3	2	3	2	2	1	2	3	2	0	2	1	33	759
(5)	2	0	1	2	X	3	2	2	3	3	2	0	2	2	1	3	1	2	31	1116
(6)	1	1	0	2	2	X	2	3	2	2	3	1	2	1	1	1	1	1	26	884
(7)	1	2	0	1	2	2	X	1	2	1	2	1	3	3	3	1	0	0	25	800
(8)	1	1	0	1	1	1	1	X	0	1	1	2	1	3	2	2	0	0	18	522
(9)	0	0	0	1	2	1	1	1	X	2	0	0	2	2	1	2	0	1	16	400
(10)	1	0	0	1	3	2	2	2	3	X	3	0	2	2	2	3	3	3	32	1120
(11)	2	1	2	1	2	1	3	1	0	1	X	1	1	1	1	1	1	1	21	735
(12)	3	1	2	2	2	3	2	3	0	3	3	X	2	2	0	0	2	0	30	360
(13)	1	1	2	2	2	1	1	1	0	1	2	1	X	1	2	2	0	3	23	805
(14)	0	0	0	1	2	2	2	3	2	2	2	0	2	X	3	3	1	0	25	750
(15)	0	0	0	1	3	1	3	1	3	3	1	0	3	3	X	3	0	0	25	600
(16)	1	0	0	1	3	3	2	1	3	3	1	0	2	2	2	X	0	2	26	598
(17)	0	0	2	1	2	1	1	0	0	3	2	0	1	0	0	0	X	0	13	208
(18)	0	0	0	2	1	2	0	1	2	3	2	0	2	0	0	2	0	X	17	238
PS	18	12	15	23	36	34	32	29	25	35	35	12	35	30	24	23	16	14		
Q	167	267	167	143	86	76	78	62	64	91	60	250	66	83	104	113	81	121		

0: Negligible effect, 1: Under proportional effect, 2: Proportional medium effect, 3: Proportional strong effect

*AS* active sum, *PS* passive sum, *P* P-value, *Q* Q-value [48]

Numbers in parenthesis indicates the serial number of the factors: leadership quality (1), building trust (2), motivation for teamwork (3), adequate knowledge (4), adequate human resources (5), capacity-building (6), shared vision and objectives (7), improving decision-making capacity (8), improving laboratory capacity (9), strengthening surveillance system (10), coordinating roles (11), relationship among actors (12), common platform (13), structured guidelines/policy (14), political will (15), specific budget head (16), community participation (17), smart technology (18)

**Fig. 1:**
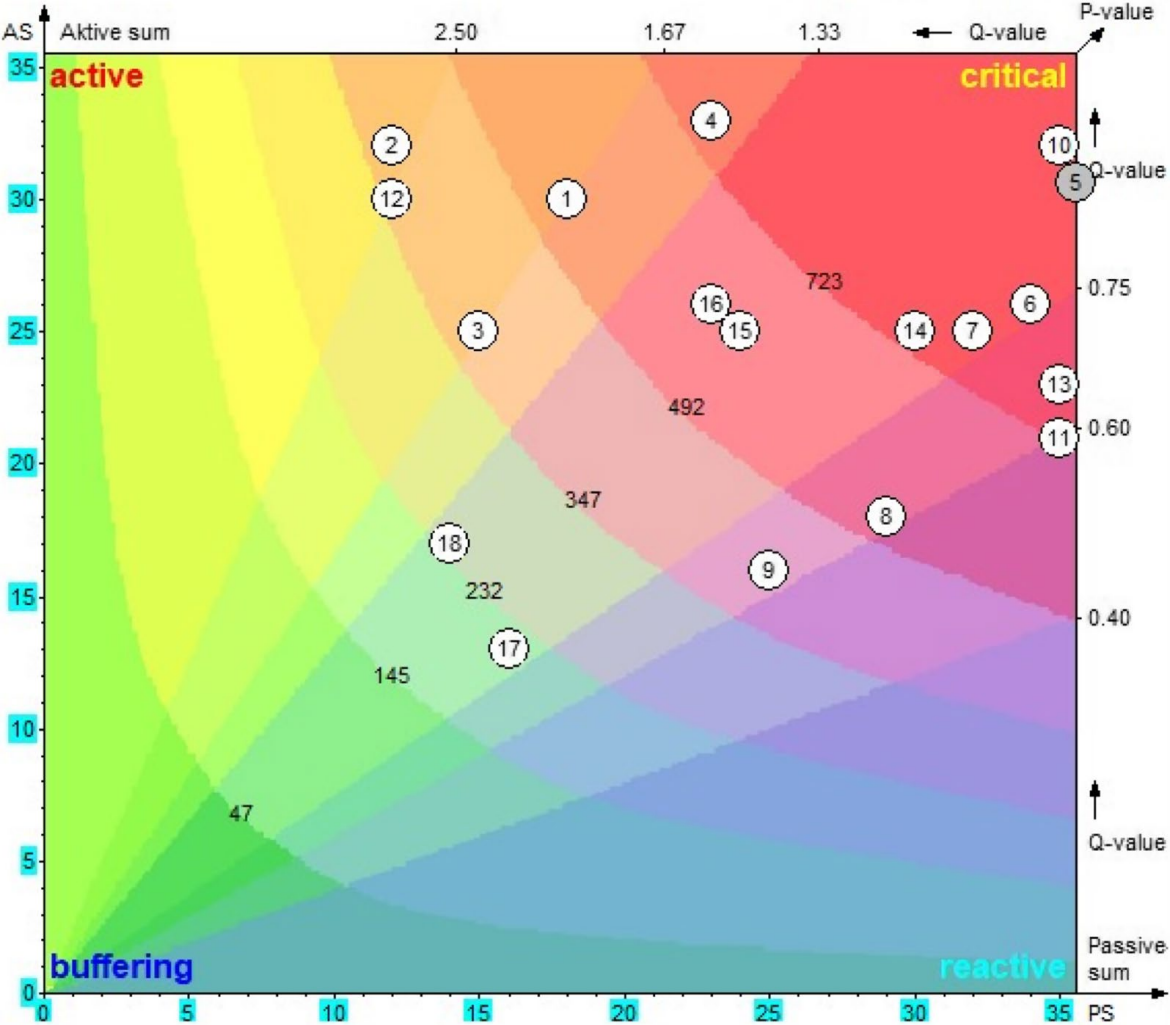
Diagram representing the systemic roles of the factors of validated OH strategies in Ahmedabad, India, extracted from the workshop during September 2019. AS: active Sum; PS: passive Sum; numbers in the circle indicates the serial number of the factors: leadership quality (1), building trust (2), motivation for teamwork (3), adequate knowledge (4), adequate human resources (5), capacity-building (6), shared vision and objectives (7), improving decision-making capacity (8), improving laboratory capacity (9), strengthening surveillance system (10), coordinating roles (11), relationship among actors (12), common platform (13), structured guidelines/policy (14), political will (15), specific budget head (16), community participation (17), smart technology (18)

The systematic role of the factors was calculated (P-value and Q-value), and the system role was assigned based on those values (as described in the method section). Figure 1 represents a geometric visualization and interpretation of each factor within the system, based on the P, Q, AS, and PS values. Each factor's role can be ascertained from the respective position in the system, as shown in Fig. 1.

All the factors are classified according to their character or systemic role into four categories: passive, active, critical, and buffering. Table 4 presents the systemic role of the factors based on P- and Q-value. Based on the Q-value, the factors are classified as either active or passive role; i.e., factors having a large quotient Q-value (e.g., “Building trust (2)” = 2.67), meaning that they have an impact on the system as they influence the system directly if the changes are considered as being active. On the other side, they cannot be steered or changed by other factors in the system. If the quotient is small, the factors are called passive (e.g., “Coordinating roles (11)” = 0.60), characterized by a reactive nature as many factors in the system influence them. Similarly, based on the P-value, the factors are assigned as either critical or buffering roles. The large product value indicates that they not only influence many other factors but, at the same time, many factors influence them (e.g., “Strengthening surveillance system (10)” = 1120). The factors with smaller product values indicate that they neither influence others nor do others influence them (e.g., “Community participation (17)” = 208). Intervention on these factors is decided based on their role in the system and the P- and Q-value. The factors with high P- and Q-values are suitable as leverage, such as “Leadership quality (1)”, as they have a salient position within the system, whereas “Community participation (17)” with low P- and Q-value is likely to be less important for this specific system functioning. However, the factors with a high P-value and a low Q-value should not necessarily be less considered, because they are strongly interwoven and have a buffering function in the system. Further, each factor's systemic role is considered with its combined effect from active–passive and critical–buffering, such as active–slightly critical, highly active–slightly critical.

**Table 4:** Systemic role of the factors based on the P-Value and Q-Value extracted from the workshop during September 2019

Active–passive	*Q*-value	Critical-buffering	*P*-value
**Highly active**		**Highly critical**	
(2) Building trust	2.67	(10) Strengthening surveillance system	1120
(12) Relationship among actors	2.50	(5) Adequate human resources	1116
**Active**		(6) Capacity building	884
(3) Motivation for teamwork	1.67	(13) Common platform	805
(1) Leadership quality	1.67	(7) Shared vision and objectives	800
**Slightly active**		(4) Adequate knowledge	759
(4) Adequate knowledge	1.43	(14) Structured guidelines/policy	750
**Neutral**		(11) Coordinating roles	735
(18) Smart technology	1.21	**Critical**	
(16) Specific budget head	1.13	(15) Political will	600
(15) Political will	1.04	(16) Specific budget head	598
(10) Strengthening surveillance system	0.91	(1) Leadership quality	540
(5) Adequate human resources	0.86	(8) Improving decision-making capacity	522
(14) Structured guidelines/policy	0.83	**Slightly critical**	
(17) Community participation	0.81	(9) Improving laboratory capacity	400
(7) Shared vision and objectives	0.78	(2) Building trust	384
(6) Capacity building	0.76	(3) Motivation for teamwork	375
**Slightly passive**		(12) Relationship among actors	360
(13) Common platform	0.66	**Neutral**	
(9) Improving laboratory capacity	0.64	(18) Smart technology	238
(8) Improving decision-making capacity	0.62	**Slightly buffering**	
(11) Coordinating roles	0.60	(17) Community participation	208

Q-Value = ASi/PSi; *P*-value = ASi*PSi

Q-value ranges: highly active (*Q* > 2.25), active (1.60 < *Q* > 2.25). moderately active (1.30 < *Q* > 1.60). neutral (0.75 < *Q* > 1.30), moderately reactive (0.60 < *Q* > 0.75), reactive (0.45 < *Q* > 0.60)., highly reactive (*Q* < 0.45)

P-value ranges: highly critical (*P* > 2.5a), critical (1.70a < *P* > 2.5a), moderately critical (1.20a < *P *> 1.70a), neutral (0.80a < *P* > 1.20a), moderately buffering (0.51a < *P* > 0.80a), buffering (0.16a < *P* > 0.50a), and highly buffering (*P* < 0.16a); where a = (n-1), *n* number of factors

#### Potential leverages of the health system (active roles)

Five factors have active roles with different ranges (highly active, active, slightly active) in the system. Of these, a micro-level factor from the individual level, i.e., “Building trust (2)” and a meso-level factor from the system level, i.e., “Relationships among actors (12)”, were observed as highly active. The other three factors having an active role were micro-level factors, i.e., “Leadership quality (1)” and “Motivation for teamwork (3)” being active, and “Adequate knowledge (4)” slightly active. This indicates that these five factors have the strongest leverage on the system and impact several other factors. However, the systemic effect was observed in combination with the role within the critical-buffering distinction. As the “Building trust (2)”, “Motivation for teamwork (3)”, and “Relationships among actors (12)” belong to the category of slightly critical, indicating that other factors influence them minimally. In contrast, “Leadership quality (1)” belongs to critical, and “Adequate knowledge (4)” belongs to highly critical, indicating that other factors influence them maximally.

For example, “Building trust (2)” affects all other factors except “Smart technology (18)” because of its highly active role. In contrast, it was less influential on “Leadership quality (1)”, “Adequate knowledge (4)”, and “Shared vision and objectives (7)” because of its slightly critical role. In contrast, another example, “Adequate knowledge (4)”, could influence most of the other factors, except “Specific budget head (16)” because of its active role, and all other factors except “Motivation for teamwork (3)” influencing it, because of its highly critical role.

With these combined roles, “Leadership quality (1)” should be carefully observed, especially if modified in order to give the development a new direction. “Building trust (2)” and “Relationships among actors (12)” effects could be canalized if interventions are made here. “Motivation for teamwork (3)” is considered as a steering lever, and it should not be untouched by the repercussions of its interventions. Therefore, it should be kept under control even after its use as a lever, and “Adequate knowledge (4)” considered with hard-hitting effect. All these five factors are ideal to be considered for the intervention as most of the factors are micro-level factors at the individual level.

#### Strong catalysts of the health system (critical roles)

There were eight factors with a highly critical role, four factors with a critical role, and four factors with a slightly critical role observed. Of the eight highly critical factors, “Strengthening surveillance system (10)”, “Adequate human resources (5)”, “Capacity-building (6)”, “Shared vision and objectives (7)”, and “Structured guidelines/policy (14)” have the same time neutral role, also indicating their strong influence on most of the other factors and vice versa. Out of four with the critical role, “Political will (15)” and “Specific budget head (16)” have a similar neutral role. Therefore, these seven factors with critical and neutral effects are described here. The remaining factors with a critical role have a secondary effect of either active or passive; thus, they are considered in the respective sections accordingly. This is because each factor has one role in the dimension of active to passive and another role in the dimension of critical to buffering.

An example of a meso-level factor from the organization level is “Adequate human resources (5)”, which has an extremely critical role in the system. This is implied by the fact that it could provoke system changes, both positively and negatively, which could lead to system instability. Uncontrolled amplifying or tipping could hardly be avoided by intervening here because this factor highly influences “Capacity-building (6)”, “Improving laboratory capacity (9)”, and “Strengthening surveillance system (10)”. External factors such as “Political will (15)” and “Specific budget head (16)” also influence it. Therefore, this factor needs to be tackled with extreme caution and should only be used as an initial ignition in extremely inactive systems. Nevertheless, the existing human resource should be mobilized to develop the ISC rather than addressing the addition of human resources to the system.

The observation indicates that “Capacity-building (6)” is influencing the other meso- and macro-level factors, while “Shared vision and objectives (7)” is influencing the external factors only. By intervening with “Strengthening surveillance system (10)”, the effect will be similar to the other meso-level factors. However, as most external factors influence it, it might require extensive resources during the intervention; thus, it is suggested to consider it at the later phase of the operationalization. The other micro- and meso-level factors influencing the three external factors are “Structured guidelines/policy (14)”, “Political will (15)”, and “Specific budget head (16)”; therefore, it is essential to be careful while addressing these factors during the operationalization process. Thus, interventions on these factors will lead to the improvement of the development of ISC; however, the absence will not make the process impossible. Although these three external factors are important and their intervention may cause trouble in the existing system, due to their equally strong activity and reaction, it has been suggested (as per the outcome of the sensitivity model) that if not intended to give a strong initial impact, they should to be targeted at a later phase of ISC development.

#### Ideal factors to monitor the health system development (reactive roles)

There were four factors, two meso-level factors from the organizational level, “Improving decision-making capacity (8)” and “Improving laboratory capacity (9)”, and two macro-level factors from the system level, “Coordinating roles (11)” and “Common platform (13)”, observed under reactive or passive roles. Out of these four critical roles, “Improving decision-making capacity (8)” has a critical role, “Improving laboratory capacity (9)” and “Relationships among actors (12)” have slightly critical roles, and “Common platform (13)” has a highly critical role. As a combined effect, other factors highly influence them in the system, and these factors have minimal influence capacity on others. Only “Common platform (13)”, with its highly critical role, could influence most of the factors with minimal strength, while other factors have a weak influencing capacity on other factors in the system.

For example, many factors influence “Common platform (13)” because of its critical role and may be able to influence factors like “Smart technology (18)”, “Motivation for teamwork (3)”, “Adequate knowledge (4)”, and/or “Coordinating roles (11)” with its highly critical role. In contrast to the previous example, most factors influence “Improving laboratory capacity (9)”, whereas it could not influence any other factors with its slightly critical role.

Effective intervention with “Improving decision-making capacity (8)” suggests that it can engender considerable changes in the system and become unmanageable by strong repercussions from the system. The role of “Improving laboratory capacity (9)” suggests that it can induce moderate changes in the system; however, it is more influenced by the other factors' effects from each level of the system. As intervention at this factor might require extensive resources, it should be entertained at the later phase of ISC development. The role of “Coordinating roles (11)” and “Common platform (13)” implies that they can incite profound changes in the system, but their effects can be slightly reinforced or weakened. The micro-level factors highly influence these two factors; thus, intervening in the micro-level factor could bring some changes to these factors during the operationalization.

#### Important factors in stabilizing the health system (neutral and buffering roles)

The role of the two external factors, “Community participation (17)” and “Smart technology (18)”, are considered as important system stabilizers. “Community participation (17)” is slightly reactive and weakly buffering, contributing to the system's self-regulation without being an indicator. The neutral factor “Smart technology (18)” has little effect on steering the system, although it is well fitted for self-regulation. Other factors have the least influence on these two factors and have minimal influence on the system's other factors. Thus, intervention on these two factors during the OH operationalization is not very beneficial for the development of ISC.

### The intervention of enabling factors for the identified OH strategies

Considering the enabling factors and their systemic role with their impact, the validated OH strategies could be achieved in two ways. One is by intervening in all factors (except buffer) and indirectly achieving the OH strategies; the other is directly achieving OH strategies individually as per the priority. As seen in Table 1, each OH strategy has its factors and it has found that “Smart technology (18)”, with its highest frequency, required for most of the strategy. However, the systemic role factor “Smart technology (18)” is observed with a neutral role signifying its presence with no effect, indicating that without this factor, the respective strategies could be achieved. In contrast, micro-level factors like “Motivation for teamwork (3)”, which is a leverage for the system with its active role and is only required to fulfil a few strategies, and another micro-level factor, “Adequate knowledge (4)”, which also has an active role, are essential for achieving most of the OH strategies. This points out that intervening with the factors with the active role is necessary as per the Vester sensitivity model; however, the least-frequency factors cannot be ignored for the intervention.

## Discussion

This paper ascertains individual factors (as active factors) are more imperative as compared to the political/external, economic, or system/network factors for the operationalization process of OH. As mentioned in the literature [51], the *individual factors* that support successful ISC for OH operationalization are education, training, prior experience, and existing relationships, whereas this study adds more in the perspective of managerial enablers, such as trust, leadership, and motivation along with subject knowledge. Similarly, the *organizational factors* already mentioned in the literature are organizational structures, culture, human resources, and communication. In addition, this study highlights capacity-building, shared vision/objectives, decision-making capacity, and adequate human resources. Evidence also indicates the *network factors* such as network structures, relationships, leadership, management, available and accessible resources, and political environment. In contrast, this study adds further factors: the coordinated roles and a common platform including the relationships with actors. Also, there are certain *external and political factors* such as structured guidelines/policy, a specific budget, strengthening laboratory and surveillance systems, the inclusion of smart technology, and last but not least, community participation and political will. In some countries where the One Health approach has been initiated, the key factors that have been discussed were political will, resources, context, common goals, strong governance, routine coordination/communication, and strong sectoral systems [6, 17, 18, 52, 53].

A cross-case analysis by Rubin et al. suggests that OH operationalization entails team-building challenges [54], and this study supports this by emphasizing individual factors as active factors that assist in successful team-building activities. Thus, a successful One Health approach will require team-building skills as fundamental core competencies. In the same line, system thinking also urges transformational leadership as an essential and prime strategy for health system strengthening [55, 56]. Similarly, in the literature, it has been documented that systemic or adaptive leadership is one of the prime necessities for any organizational cultural model [57]. An interpretive study by Wong et al. identified systemic factors for ISC as structures, funding models, regulatory policies, power relations, harmonized information and communication infrastructure, targeted professional education, and formal systems leaders as collaborative champions [58], which also became evident in our findings. Another review argues that for effective implementation, lessons learned and “best practice” must be led by regional stakeholders drawn from a variety of disciplines [59]; that means the local actors are more influential in OH operationalization. The factors that have emerged for operationalizing OH from the local stakeholders were based on their experience and expertise in the respective sectors. ISC is rarely without complications; however, drawing shreds of evidence from the local actors with the identified strategies and enabling factors will smoothen OH's operationalization process.

This case study is unique in revealing the importance of the local stakeholders and the bottom-up approach, which are more appropriate strategies to the concerned health system at the operational level. On the one hand, external factors like political will and a specific budget are important influencers for the operationalization of OH. On the other hand, the micro-level factors at the individual level, like trust, leadership, and motivation, are essential drivers at the grass-roots level. This system approach analysis strongly recommends that the OH operationalization at the grass-roots level could be initiated by innerving the factors with an active role, i.e., most of the micro-level factors identified in the study, except motivation for teamwork. Additionally, addressing the other macro-level factors with an active role in the system, e.g., instituting relationships among actors, will also enhance this operationalization process. As most of the external factors are critical or neutral, the immediate intervention should not target these factors. In the longer term, once the micro-, meso-, and macro-level factors are strengthened and stabilized, addressing the external factors is recommended. As the meso-level factors are highly influenced by either micro or external factors, it is recommended to address the micro-level factors during the initial phase as these are found to have an active role in the system. In addition, most of the micro-level factors could be intervened with minimal cost and thus be supportive in addressing the preliminary phase of operationalization. In general, the collectivistic leadership in healthcare has demonstrated a positive impact, according to recent implementation health research [60, 61]. The special requirements of OH operationalization also endorse the strengthening of collaborative, transformational conflict management leadership development across OH actors [62]. This case study unfolds the importance of the system approach in identifying the local health system's needs. Although this case study emphasizes the local health system for OH's operationalization, similar kinds of research are recommended to understand the scenarios for the regional, national, and global needs. The future OH policies should prioritize balance between the subject knowledge development and the OH actors' leadership competencies, which becomes a prime goal for OH operationalization.

With the current ongoing pandemic of COVID19, the method adopted in this study, i.e., the systems approach, could be advantageous to policy-makers in understanding the spread of infection and its multifaceted consequences among the community, as society is itself a complex adaptive system [63, 64]. As compared to the linear problem-solving approach, this systems approach to pandemic prevention goes beyond this interface and includes an understanding of environmental drivers and the socio-ecological context of disease emergence. This entails addressing essential issues of other integrated systems such as the education system, transport system, food system, and many more to tackle pandemics' underlying causes [65]. It is equally important to consider the bottom-up approach, which provides new insight into the ISC development and indicates the importance of the micro-level factors at the individual level over the other enabling factors for OH operationalization. Thus, the bottom-up approach remained an utmost important exploration in operational research, especially for the local health system. This approach could be highly beneficial to develop strategies where policy is absent.

## Conclusion

The operationalization of collaborative preventive strategies of OH relies on the full adhesion to necessary micro-level factors at the individual level followed by the macro- and meso-level factors. The willingness of actors to embark on this resource-consuming collaborative strategy depends on the relationship among staff and the trust with other sectors, followed by leadership quality and staff motivation. Additionally, external factors, such as structured guidelines and political will, are needed but not vital as micro-level factors to initiate the ISC. This study provides insight into the type of enabling factors, which could be actively addressed through adequate intervention without affecting the health system's resilience during the operationalization process. The system approach through a bottom-up exploration is recommended to understand the local health system and its enabling factors during ISC development as part of OH operationalization.

## Supplementary Information

**Additional file 1:** RICOHA Form-5.1: Interview guide for vignette study.

**Additional file 2:** RIOCHA Form-5.2: Policy Delphi tool for validation of key strategies.

**Additional file 3:** RICOHA Form-5.3: Semi-structured tool for the participatory system workshop.

## Data Availability

All relevant data that supports the findings of this study are within the manuscript.

## Abbreviations

AS: Active sum
ISC: Intersectoral collaboration
OH: One Health
PS: Passive sum
RICOHA: Research to explore intersectoral collaboration for One Health approach
