# Supplementary material for: Systemic factors for enhancing intersectoral collaboration for the operationalization of One Health: a case study in India
Source: Health Res Policy Syst. 2021 May 4;19:75. doi: 10.1186/s12961-021-00727-9 (PMC8097865; doi:10.1186/s12961-021-00727-9)
Supplement: Supplementary file 1 — Additional file 1: RICOHA Form-5.1: Interview guide for vignette study. [file 12961_2021_727_MOESM1_ESM.pdf]

Additional file 1. (RICOHA form 5.1: Interview guide for Vignette study)

ONE HEALTH
& URBAN TRANSFORMATION

Topic: General and personal information

1. Introduction of the research and agreement for the interview.

2. What is your experience in the prevention & control of zoonotic diseases?

Topic: Convergence pattern

[Brief about the importance of One Health Collaboration]

3. In your opinion, when the actors from the human and the animal health system should collaborate for effectual prevention and control of zoonoses?

4. If we consider the three-tier health system of India, in your opinion, where the convergence of actors from the human and the animal health system need to be focused? [Prompt: Entry points for collaboration]

5. In your view, how could the collaboration between the human and the animal health system be strengthened? [Prompt: Strategies for collaboration (early detection, combined health services), Ways to engage private actors]

6. In your opinion, what should be the ideal reporting pattern for the early detection of zoonotic diseases among key actors of the human & animal health system?

7. In your opinion, what should be the ideal roles & responsibilities of the following actors with reference to the collaboration?

    a. Actors at the community level
    b. Actors at the clinical level
    c. Actors at the managerial level

8. What needs to be done further to strengthen the collaboration between various actors?

[Prompt: Consider for disease-specific i.e., Rabies, Brucellosis, Influenza (H1N1, H5N1) and at different levels of health system]

Any further suggestions!
