# Supplementary material for: Systemic factors for enhancing intersectoral collaboration for the operationalization of One Health: a case study in India
Source: Health Res Policy Syst. 2021 May 4;19:75. doi: 10.1186/s12961-021-00727-9 (PMC8097865; doi:10.1186/s12961-021-00727-9)
Supplement: Supplementary file 2 — Additional file 2: RIOCHA Form-5.2: Policy Delphi tool for validation of key strategies. [file 12961_2021_727_MOESM2_ESM.pdf]

Additional file 2. (RIOCHA Form-5.2: Policy Delphi tool for validation of key strategies)

[Online survey through Survey monkey]

**Section-1: Basic information**			
**UID**			
**Name**			
**Department**		**Designation**	
**Mobile**		**GPS**	
**Completed age (in years)**			
**Gender**			
**Highest educational qualification**			
**Total years of experience (in years)**			

Section-2: Key strategies on effectual prevention and control of zoonotic diseases in Ahmedabad, India

**Strategies**	**Somewhat preferable**	**Very much preferable**	**Somewhat essential**	**Very much essential**
**Legal strategies or policies**				
Public health act or clinical establishment act for all the clinics in the state emphasizing reporting any conditions to the public health system	①	②	③	④
Guidelines for disposal of all dead animals irrespective of the disease condition	①	②	③	④
Development of joint guidelines (clinical/preventive) for each prioritized zoonotic diseases	①	②	③	④
Urban city should have animal treatment centers, hostel facility where stray animals can be inspected and vaccinated is recommended	①	②	③	④
Bi-directional and cross-flow of information is recommended with respective departments	①	②	③	④

Zoonoses committee at district and state should act as the prime platform for One Health and similar zoonoses committee is encouraged at the urban setting too.	①	②	③	④
Provision of Animal Health Card/Passport for animals and compelling to use while buying/selling any animals	①	②	③	④
**Clinical aspects or disease-specific**				
Strengthening the capacity of laboratories for screening and diagnosing zoonotic conditions	①	②	③	④
Prophylactic vaccination of animals for rabies prevention	①	②	③	④
Capacity development for differential diagnosis of brucellosis as an ultimate prevention strategy	①	②	③	④
Promoting good hygiene for flu prevention	①	②	③	④
**Collaboration at the managerial level**				
Resource sharing (especially Human resources) within each department to initiate the One Health approach	①	②	③	④
Regular data sharing and joint-data analysis	①	②	③	④
Information flowing from various departments to a single platform for early prediction of emerging diseases	①	②	③	④
Regular joint meetings among the program planners, even though there is no epidemic or out-break	①	②	③	④
Reporting pattern for prioritized zoonotic conditions to be established and regular monitoring of the same is essential	①	②	③	④
Professional bodies like IMA, GVC should initiate collaborative activities	①	②	③	④
**Collaboration at the provider level**				
Sharing of knowledge among Medical doctor and Veterinarians through a common platform	①	②	③	④
A common One Health clinical body that is answerable for every queries related to zoonoses and assist in the clinical practice.	①	②	③	④
The IEC materials should be with Medical doctors as well as Veterinarians to educate their patients	①	②	③	④
Social media like WhatsApp group of different clinical groups should be merged together for early alert system	①	②	③	④
There is a need to improve the early alert system about zoonoses much prior to the outbreak across the professionals	①	②	③	④

Joint training on Zoonoses for medical doctors and veterinarians	①	②	③	④
A detailed history taking for provisionally diagnosis of zoonotic conditions at primary care setting	①	②	③	④
There should be a system of cross-referral of cases between medical doctor and veterinarian for respective risk assessment	①	②	③	④
Symptom based early diagnosis capacity need to be developed among medical doctors and veterinarians	①	②	③	④
**Collaboration at the community level**				
One Health committee at the grass root level could be formulated with the help of the frontline workers along with the community representative	①	②	③	④
The frontline workers should communicate each other at the grass root level and also should inform both officials for any abnormal occurrence	①	②	③	④
Sensitization of community along with knowledge and awareness on prevention and control of zoonoses	①	②	③	④
Financial incentives to the animal handlers to report any disease or any abnormal condition of their animals to the system	①	②	③	④
Community awareness by the grass root healthcare workers	①	②	③	④
**Inclusion of private actors**				
Financial incentive package to private providers for reporting a zoonosis to the public health system	①	②	③	④
There should be no financial incentive for the private actors to report any zoonotic diseases, it should be by law	①	②	③	④
Dairy personnel could be trained further to improve the scope of symptom based disease detection among the animals	①	②	③	④
Sensitization and promotion of ethical practices among private providers as essential to bring them to the health system	①	②	③	④
Social media of private actors should be integrated with the public health actors, to be a potential platform to exchange the knowledge and early detection of zoonoses	①	②	③	④
