# Supplementary material for: Systemic factors for enhancing intersectoral collaboration for the operationalization of One Health: a case study in India
Source: Health Res Policy Syst. 2021 May 4;19:75. doi: 10.1186/s12961-021-00727-9 (PMC8097865; doi:10.1186/s12961-021-00727-9)
Supplement: Supplementary file 3 — Additional file 3: RICOHA Form-5.3: Semi-structured tool for the participatory system workshop. [file 12961_2021_727_MOESM3_ESM.pdf]

Additional file 3. (RICOHA form 5.3: Semi-structured tool for the participatory system workshop)

ONE HEALTH & URBAN TRANSFORMATION

Step-1: System description

Here, a system is defined as the health system of Ahmedabad city, which comprises both the human and animal health sub-systems. This also comprises of the public and the private actors from both the system.

Step-2: Defining variables

Please suggest further factors that you think would be considered while developing One Health inter-sectoral collaboration

Step-3: Criteria assignments (Criteria Matrix)

To verify their completeness (from a "systems" viewpoint), all indicators were cross-checked against the fixed criteria. These criteria define the indicators' representativeness, physical quality, dynamics, and entropy. The possible fitting scores are:

- Fully applicable (dark ● circle; 1)
- Partially applicable (open ○ circle; 0.5)
- No relevance (empty; 0)

The values for each domain are summed up and compared amongst one another, seeking a balanced coverage of all systemic aspects.

**Criteria**	**Description**
**Spheres of Life**	
Economy	Activities (What they do?) Capital production, tax receipts, debts, shareholder value
Population	Participants (Who are they all?) Number, structure and dynamics, working people, age structure
Space utilization	Space (What happens where?) Use of space, land development, residential structure
Human ecology	Mood (How do people feel?) Human ecology, social structure, quality of life, security, education, state of health
Natural balance	Natural balance (How does resources budget work?) Consumption of raw materials, energy, water, soil sealing, influence on climate
Infrastructure	Internal processes (What channels of communication are there?) Transport and access roads, tele communications, traffic and supply
Rules & Laws	Internal order (How is this regulated?) Local government, taxes, measures, ordinances and legislations, planning procedure
**Physical Category**	
Matter	Factors having a primarily material character (E.g. Buildings, raw materials, people, animals, plants, vehicles)
Energy	Factors having a primarily energy-related character (E.g. Power consumption, Workers, Energy carriers, financial strengths)

Information	Factors having a primarily information-related and communication-related character (E.g. Media, decisions, explication, exchange of information, orders, perception, acceptance)
**Dynamic Category**	
Flow quantity	Factors expressing primarily flows of matter, energy, or information within the system (E.g. Power consumption, traffic, commuters, instructions)
Structure quantity	Factors serving to determine structure rather than flow (E.g. Green spaces, population densities, traffic network, accessibility, hierarchy)
Temporal dynamics	Factors that at the same location change at a given time or that possess temporal dynamics (E.g. Seasonal activity, election meetings, climate factors, transport timetables, tax checking)
Spatial dynamics	Factors that at a given time differ from location to location (E.g. Traffic revenue, industrial effluent, nature-conservation area)
**System relationship**	
Opens the system through inputs	Factors that open the system through influences from outside (E.g. precipitation, dumping, imports, tourism)
Opens the system through outputs	Factors that open the system through influences from the inside (E.g. Waste water, commuters leaving the city, exports)
Can be influenced from inside	Factors that can be controlled by decision-making processes coming from within the system under consideration. Among other things, these are a measure of the system's self-sufficiency
Can be influenced from outside	Factors that are subject to decision-making processes taking place outside the system under consideration. Among other things, these are a measure of the system's dependence

Step-4: Matrix of Consensus (Impact Matrix)

Strength of connections should be assigned values between 0 to 3.

- **3 (Disproportionally strong connection):** If A changes only a little, B changes a lot
- **2 (Medium strength, more or less proportional connections):** If A changes a lot in order to achieve a more or less equally big change in B
- **1 (Weak connections):** If a marked change in A brings about only a weak change in B
- **0 (No connections):** No effect at all, a very weak effect or an effect occurring only after a lengthy delay

	F1	F2	F3	F4	F5
F1					
F2					
F3					
F4					
F5					
